# Hippo pathway at the crossroads of stemness and therapeutic resistance in breast cancer

**DOI:** 10.1002/1878-0261.70232

**Published:** 2026-03-26

**Authors:** Giulia Schiavoni, Antonella Palmese, Stefano Scalera, Laura Cipriani, Davide Mascolo, Patrizia Vici, Teresa Arcuri, Lorena Filomeno, Eriseld Krasniqi, Giovanni Blandino, Giulia Bon, Marcello Maugeri‐Saccà

**Affiliations:** ^1^ Clinical Trial Center, Biostatistics and Bioinformatics Unit IRCCS Regina Elena National Cancer Institute Rome Italy; ^2^ Cellular and Molecular Biology, Department of Biology University of Rome “Tor Vergata” Italy; ^3^ Cellular Network and Molecular Therapeutic Target Unit, Department of Research, Diagnosis and Innovative Technologies IRCCS Regina Elena National Cancer Institute Rome Italy; ^4^ Department of Computer, Automatic and Management Engineering (DIAG) Sapienza University of Rome Italy; ^5^ Phase IV Studies IRCCS Regina Elena National Cancer Institute Rome Italy; ^6^ Oncogenomic and Epigenetic Unit, Department of Research, Diagnosis and Innovative Technologies IRCCS Regina Elena National Cancer Institute Rome Italy; ^7^ Division of Medical Oncology 2 IRCCS Regina Elena National Cancer Institute Rome Italy

**Keywords:** breast cancer, cancer therapy resistance, Hippo pathway, stemness, triple‐negative breast cancer

## Abstract

Breast cancer, the most frequently diagnosed cancer in women globally, is a heterogeneous disease with distinct subtypes requiring distinct therapeutic approaches. Regardless of molecular subtyping, breast cancer stem cells significantly contribute to tumor heterogeneity, distant dissemination, and therapeutic resistance. The Hippo pathway is a key regulator of organogenesis and tissue development, and its deregulation is common in breast cancer and linked to cancer stem cell features across several cancer types. Dysfunctional pathway activity leads to the aberrant activation of Hippo downstream effectors, the Yes‐associated protein (YAP) and its paralog transcriptional co‐activator with PDZ‐binding motif (TAZ), which promote epithelial‐to‐mesenchymal transition, growth factor‐independent proliferation, and maintenance of the breast cancer stem cells' niche. This review summarizes the regulation of the Hippo pathway, emphasizing its significant role in coordinating stemness‐related mechanisms in breast cancer. An overview of how the Hippo pathway fuels stemness in triple‐negative breast cancer, the most aggressive BC subtype, is then provided. We also discuss how the activation of stem cell‐like properties, driven by dysregulation of the Hippo pathway, contributes to the development of resistance to current therapies across the spectrum of breast cancer subtypes.

## Graphical abstract

Dysregulation of the Hippo pathway drives nuclear accumulation of YAP/TAZ, activating stemness‐related transcriptional programs that sustain breast cancer stemness and fuel therapeutic resistance across subtypes, underscoring Hippo signaling as a targetable vulnerability. Figure created and edited with BioRender.com.

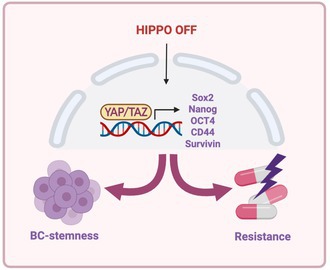

**Abbreviations**
ADC: antibody‐drug conjugate
Akt: AKT serine/threonine kinase 1
ALDH1: aldehyde dehydrogenase 1
AMOTL1: Angiomotin like 1
Amot‐p80: Angiomotin p80
AMPK: AMP‐activated protein kinase
ANKRD1: ankyrin repeat domain 1
BC: breast cancer
BCSCs: breast cancer stem cells
BRD4: bromodomain‐containing protein 4
CCDC80: coiled‐coil domain‐containing 80
CCL2: chemokine (C‐C motif) ligand 2
CCR2: C‐C chemokine receptor 2
Cdk1: cyclin‐dependent kinase 1
CDK4/6: cyclin‐dependent kinase 4 and 6
CDK7: cyclin‐dependent kinase 7
CDK9: cyclin‐dependent kinase 9
CPZ: chlorpromazine
CSCs: cancer stem cells
CTGF: connective tissue growth factor
CYR61: Cysteine‐rich angiogenic inducer 61
DDR: DNA damage response
ECM: extracellular matrix
EMT: epithelial‐to‐mesenchymal transition
ER: estrogen receptor
ERK: extracellular signal regulated kinase
F3: Coagulation factor III
FAT1: FAT atypical cadherin 1
FOXM1: forkhead box protein M1
GADD45A: growth arrest and DNA damage inducible alpha
GAP: GTPase‐activating protein
GPCR: G‐protein‐coupled receptors
GPER: G‐protein‐coupled estrogen receptor
GRK3: G protein‐coupled receptor kinase 3
HDAC: histone deacetylase
HER2: human epidermal growth factor receptor 2
HMG‐CoA: 3‐hydroxy‐3‐methyl‐glutaryl coenzyme A
HR: hormone receptor
IGF‐1: insulin‐like growth factor 1
IGF‐1R: insulin‐like growth factor 1 receptor
IL‐6: interleukin 6
LATS1/2: large tumor suppressor 1 and 2
LEF: lymphoid enhancer factor
LIFR: leukemia inhibitory factor receptor
lncRNA: long non‐coding RNA
MAPK: mitogen‐activated protein kinase
MEKK3: mitogen‐activated protein kinase kinase kinase 3
miRNA: microRNA
MOB1A/B: MOB kinase activator 1A and 1B
MST1/2: mammalian sterile 20‐like kinase 1 and 2
mTOR: mammalian target of rapamycin
mTORC1: mammalian target of rapamycin complex 1
MYH9: myosin heavy chain 9
NANOG: homebox protein NANOG
NCoR2: nuclear receptor corepressor 2
NF2: neurofibromin 2
OTUD5: OUT deubiquitinase 5
PD‐L1: programmed death‐ligand 1
PDX: patient‐derived xenograft
PI3K: phosphoinositide 3‐kinase
RAD18: RAD18 E3 ubiquitin protein ligase
RhoGAP: interacting with CIP4 homologs protein 1
RICH1: RhoA: Ras homolog family member A
ROCK: Rho‐associated protein kinase
ROR1: type 1 receptor tyrosine kinase‐like orphan receptor
RUNX1: Runt‐related transcription factor 1
RUNX2: Runt‐related transcription factor 2
SAV1: Salvador homolog 1
Scribble: Scribble planar cell polarity protein
SERM: selective estrogen receptor modulator
SIAH2: seven in absentia homology 2
SMAD: small mother against decapentaplegic
SMARCA4: SWI/SNF‐related BAF chromatin remodeling complex subunit ATPase 4
SMRT: silencing mediator for retinoic acid and thyroid hormone receptors
SOX2: stemness‐related sex‐determining region Y‐box 2
SRF: serum‐response factor
STAT3: signal transducer and activator of transcription 3
SWI/SNF: SWItch/Sucrose Non‐Fermentable
TAM: tumor‐associated macrophages
TAZ: transcriptional co‐activator with PDZ‐binding motif
TBX5: T‐box transcription factor 5
TCF: T‐cell factor
TCGA: The Cancer Genome Atlas
TEAD: TEA domain‐containing sequence‐specific transcription factors
TGFβ: transforming growth factor β
TME: tumor microenvironment
TNBC: triple‐negative breast cancer
TRIM58: tripartite motif containing 58
VGLL3: vestigial‐like protein 3
WWOX: WW domain containing oxidoreductase
YAP: Yes‐associated protein
YTHDF1: YTH N6‐methyladenosine RNA binding protein F1
ZO‐2: zona occludens‐2

## 1 Introduction

Breast cancer (BC) is the most frequently diagnosed cancer in females worldwide [1]. Advances in early detection and targeted treatments over recent decades have significantly reduced BC mortality rates [2]. Based on the expression of established biomarkers—estrogen receptor (ER), progesterone receptor (PR), and human epidermal growth factor receptor 2 (HER2)—four primary molecular subtypes are recognized: luminal A (ER/PR‐positive, HER2‐negative); luminal B (ER/PR‐positive, HER2‐negative or positive; more aggressive than luminal A and differing for higher levels of ki67); HER2‐enriched (ER‐negative, PR‐negative, HER2‐positive); and triple‐negative breast cancer (TNBC; ER‐negative, PR‐negative, HER2‐negative). These subtypes exhibit distinct biological and clinical behaviors, requiring tailored therapeutic approaches. More recently, the HER2‐low context has provided a further level of resolution to the biology of BC [3].

Cancer stem cells (CSCs) are extensively studied due to their significant contribution to tumor heterogeneity, therapeutic resistance, tumor recurrence, and metastasis. BC stem cells (BCSCs) were first identified by Al‐Hajj and colleagues as tumorigenic cells characterized by the CD44^+^/CD24^−/low^ phenotype. These cells demonstrated self‐renewal potential, extensive proliferation, and the ability to differentiate into heterogeneous mature cell types *in vitro* [4]. Further characterization revealed high aldehyde dehydrogenase 1 (ALDH1) enzyme activity, which enables the inactivation of chemotherapeutic agents and enhances DNA damage response (DDR) pathways [5, 6, 7]. BCSCs are also associated with distant dissemination and poor clinical outcomes across BC subtypes. For instance, CD44^+^/CD24^−/low^ cells exhibit mesenchymal and quiescent phenotypes, while ALDH1^+^ cells display epithelial and proliferative traits, reflecting distinct roles in metastasis and localized growth [8, 9, 10]. A substantial body of evidence has clarified the specific molecular mechanisms underlying the contribution of BCSCs to therapeutic resistance in different subtypes of BC, including dysregulated PI3K/AKT/mTOR, Notch, and Wnt/β‐catenin signaling, stemness transcriptional networks, and microenvironmental interactions that promote survival under endocrine, HER2‐targeted, and chemotherapeutic pressures [11, 12, 13, 14, 15, 16, 17]. TNBC, enriched with BCSCs, shows the highest sphere‐forming capacity and stemness gene signatures (e.g., SOX2, OCT4), which correlate with aggressive behavior and resistance to conventional therapies [18].

Among the variety of molecular deregulations in BC, the Hippo pathway plays a critical role in the acquisition of stemness‐related properties. As initially demonstrated by Cordenonsi and colleagues, dysregulation of Hippo pathway components occurs early during transformation and controls epithelial‐to‐mesenchymal (EMT) transition, growth factor‐independent proliferation, and focal adhesion formation [19]. Pathway dysregulation is associated with poorer prognosis and metastatization [20]. Importantly, the Hippo pathway maintains the BCSC niche by regulating stemness. Impaired Hippo signaling correlates with enrichment of the CD44^+^/CD24^−/low^ cell population, driven by yes‐associated protein (YAP)‐induced transcription of stemness‐related genes and results in therapeutic failure through subtype‐specific mechanisms of action [21, 22, 23, 24].

On this ground, the aim of this review is to provide an overview of the Hippo pathway's role in coordinating the interplay between stemness‐related mechanisms and the onset of therapeutic resistance in BC.

## 2 The Hippo pathway

**Fig. 1:**
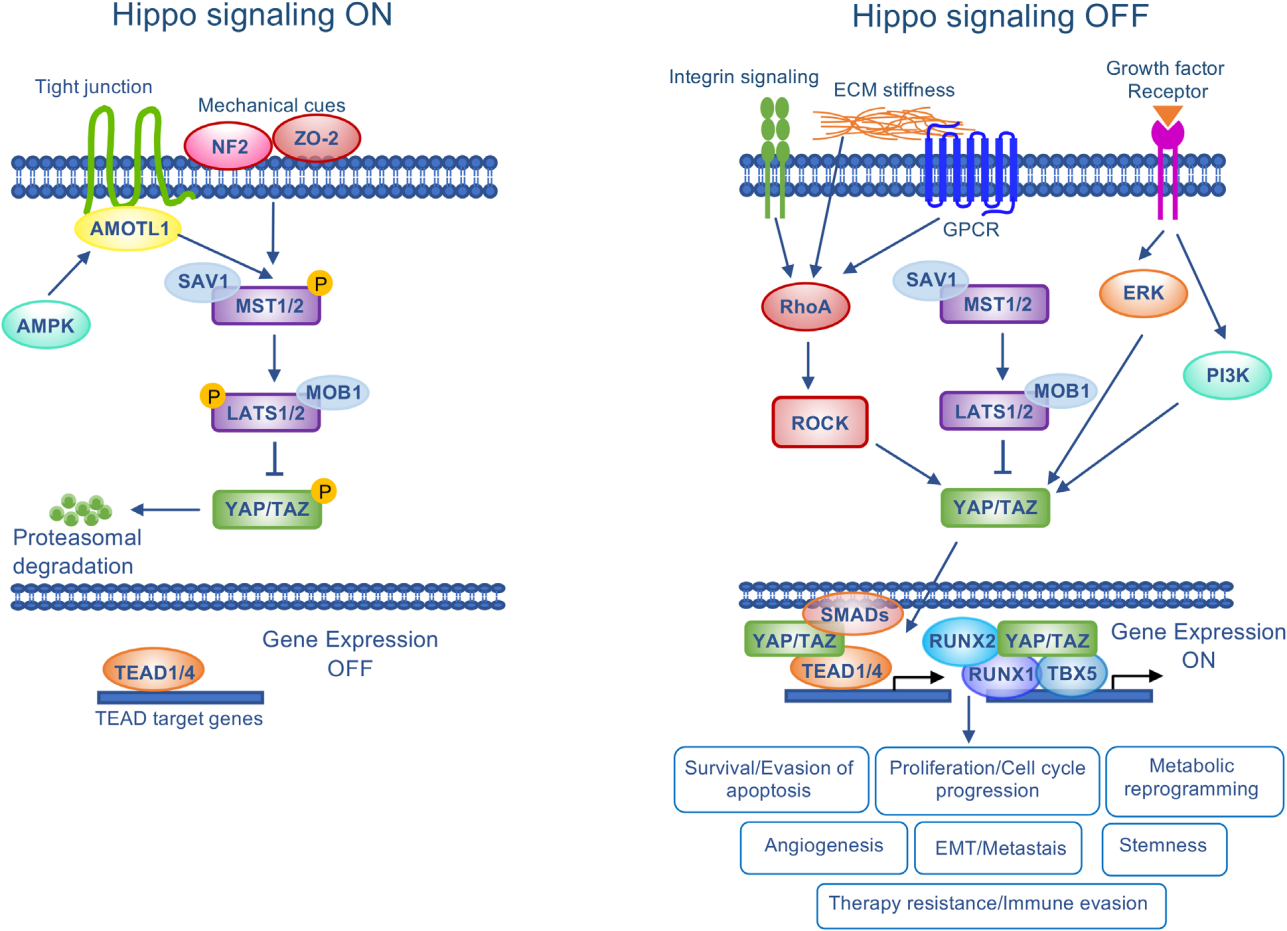
Hippo signaling pathway and its regulatory network. The Hippo pathway is activated by upstream signals (Hippo ON) that enable the phosphorylation resulting in YAP/TAZ proteasomal degradation (leftmost panel). Mechanical signaling, hormones and growth factors promote YAP/TAZ translocation into the nucleus (Hippo OFF, rightmost panel). Here, YAP/TAZ interact with TEAD as well as with SMADs, TBX5, RUNX1, and RUNX2 transcription factors and co‐factors to activate the transcription of target genes and drive pro‐tumorigenic processes.

The Hippo pathway is an evolutionary conserved signaling cascade involved in the balanced control of organogenesis and tissue development. Initially identified in *Drosophila melanogaster*, its role was later confirmed in mammals, including mice [25, 26]. Mutagenesis screening‐based studies in *Drosophila* led to the identification of the *wts* tumor suppressor gene as a key modulator of cell proliferation during development [25]. Following the identification of key pathway components, Dong and colleagues developed a YAP transgenic mouse model, which was instrumental in identifying YAP as the nuclear effector of the Hippo pathway and confirming its role in mammalian organ size control and tumorigenesis [26]. A number of subsequent studies [27, 28, 29, 30, 31, 32] clarified the organization of the pathway along with multiple intersecting mechanisms that either activate or inhibit various pathway components. Shortly, the Hippo pathway is regulated by a variety of stimuli, including: (i) mechanical cues, which can be mediated by adherents junctions, cytoskeleton remodeling, or the action of proteins such as Neurofibromin‐2 (NF2) or Zona occuldens (ZO); (ii) cell surface receptors such as the G‐protein‐coupled receptors (GPCR) or (iii) crosstalk with other signaling pathways including mammalian target of rapamycin (mTOR), stress signals, and metabolic pathways such as those involving glucose, fatty acids, the mevalonate pathway and the AMP‐activated protein kinase (AMPK) pathway [33, 34, 35, 36]. The Hippo pathway core consists of several serine/threonine kinases that activate a phosphorylation cascade. Mammalian sterile 2‐like kinase1 and 2 (MST1/2) bind to Salvador homolog 1 (SAV1), inducing the phosphorylation of large tumor suppressor 1 and 2 (LATS1/2). The binding with their cofactor MOB kinase activator 1A and 1B (MOB1A/B) allows the phosphorylation of YAP/TAZ, resulting in their cytoplasmic retention and proteasomal degradation [33, 37]. When Hippo signaling is inactivated, YAP and TAZ can translocate into the nucleus, where they promote gene expression by interacting with Transcriptional Enhanced Associated Domain (TEAD), as well as with other transcription factors such as small mother against decapentaplegic (SMADs), T‐box transcription factor 5 (TBX5), Runt‐related transcription factor (RUNX)1 and RUNX2 [22, 38, 39, 40] (Fig. 1).

**Fig. 2:**
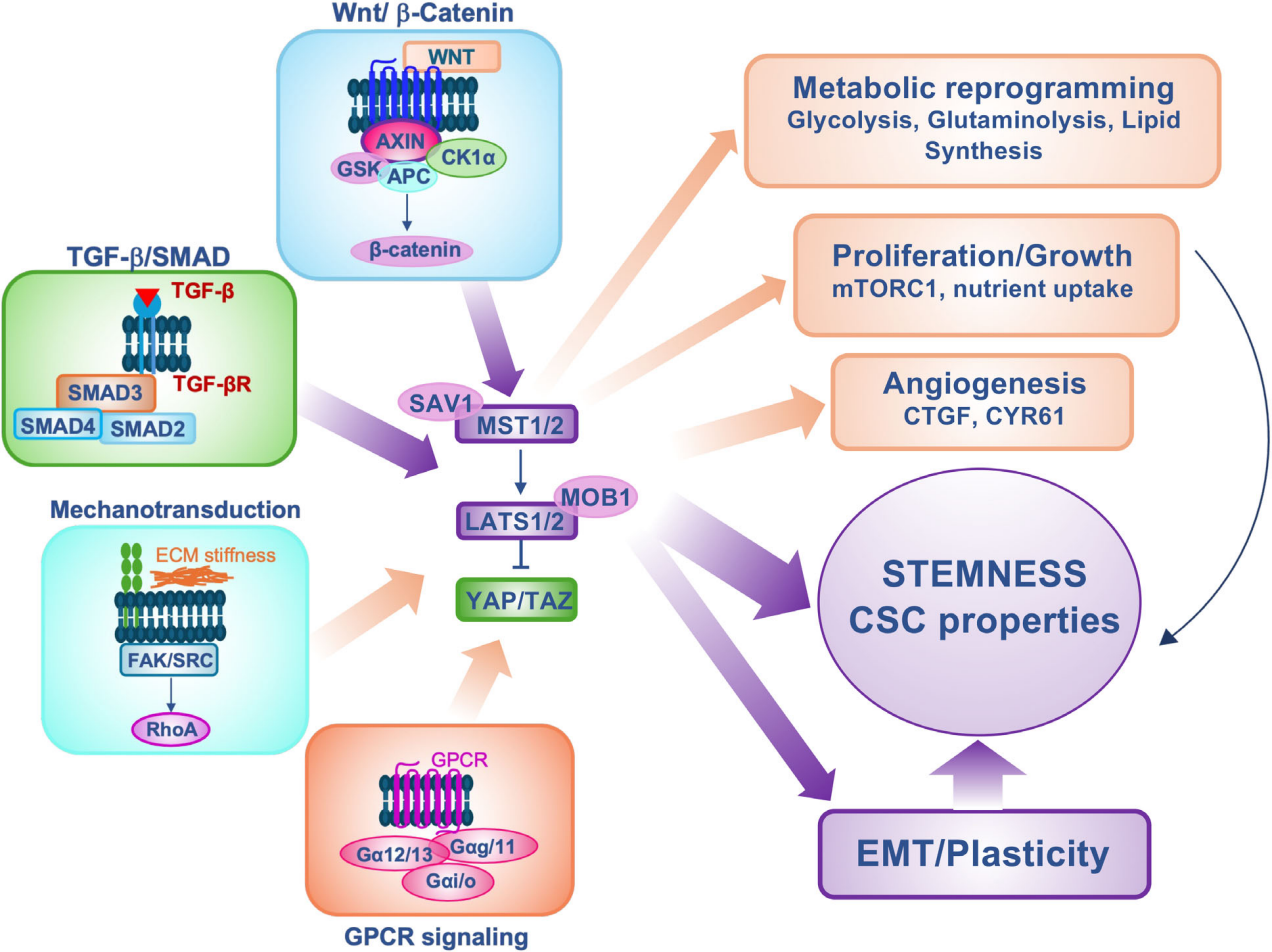
YAP/TAZ‐centered signaling networks converging on cancer stemness. YAP and TAZ act as central hubs integrating multiple upstream regulatory inputs, including Wnt/β‐catenin signaling, TGF‐β/SMAD signaling, mechanotransduction pathways, and GPCR‐mediated cues. Through direct transcriptional cooperation or indirect modulation of cellular programs such as epithelial‐mesenchymal transition (EMT) and cellular plasticity, YAP/TAZ promote the acquisition and maintenance of stem‐like traits in cancer cells. Additional YAP/TAZ‐regulated processes, including metabolic reprogramming, proliferative signaling, and angiogenesis, support stemness indirectly by sustaining the bioenergetic and anabolic requirements of cancer stem cells. Purple arrows indicate pathways more directly linked to stemness regulation, whereas apricot arrows denote indirect or supportive contributions.

Hippo signaling is often hyperactivated during tumorigenesis due to mutations in pathway components (MST1/2, LATS1/2, YAP and TAZ) [41] and/or functional deregulation of upstream or lateral regulatory mechanisms. Hyperactivation of YAP and TAZ drives cell growth and proliferation through multiple oncogenic mechanisms. These include functional interplay with the mTOR signaling pathway, where YAP/TAZ enhance mammalian target of rapamycin complex 1 (mTORC1) activity by promoting amino acid uptake and nutrient sensing, thus sustaining anabolic growth and protein synthesis [42]. In parallel, YAP and TAZ facilitate the assembly of focal adhesions by regulating the expression of cytoskeletal and adhesion‐related genes, such as those encoding integrins and vinculin, which strengthens cell‐matrix interactions and promotes migration and mechanosensing [43]. Additionally, YAP/TAZ drive the metabolic reprogramming of cancer cells through broad transcriptional regulation of genes involved in glycolysis, glutaminolysis, and lipid biosynthesis, thereby supporting the energetic and biosynthetic demands of rapidly proliferating cells [44, 45]. In addition to their canonical roles in mechanotransduction and transcriptional regulation, YAP and TAZ act as critical nodes of crosstalk with several other signaling pathways, enhancing their oncogenic potential and reinforcing cellular plasticity. In the Wnt/β‐catenin pathway, they cooperate with β‐catenin to enhance transcription of stemness‐ and proliferation‐related genes, sometimes competing for or co‐binding T‐cell factor/lymphoid enhancer factor (TCF/LEF) transcription factors, and are even required for β‐catenin nuclear localization in certain contexts, collectively amplifying oncogenic signaling [46]. In the TGF‐β/SMAD axis, YAP/TAZ interact with SMAD2/3 to co‐drive gene programs promoting EMT, immune evasion, and fibrosis, effectively shifting TGF‐β responses from tumor‐suppressive to tumor‐promoting [47, 48]. Additionally, GPCR signaling regulates YAP/TAZ through cytoskeletal dynamics and Rho GTPase activity: Gα12/13‐, Gαq/11‐, and Gαi/o‐coupled receptors activate YAP/TAZ by inhibiting the Hippo pathway, while Gαs‐coupled receptors repress them via PKA activation and LATS1/2 stimulation [35]. YAP/TAZ also facilitate tumor vascularization by upregulating pro‐angiogenic factors, notably connective tissue growth factor (CTGF) and cysteine‐rich angiogenic inducer 61 (CYR61), thereby sustaining the nutrient and oxygen demands of rapidly growing tumors [49]. Altogether, the pleiotropic actions of YAP and TAZ establish them as central orchestrators of cellular plasticity, tissue regeneration, and malignant transformation, making them attractive targets for therapeutic intervention in cancer. Importantly, YAP/TAZ act as central hubs integrating diverse signaling pathways, including metabolic rewiring, mechanotransduction, EMT, and crosstalk with Wnt and TGF‐β, to promote cellular plasticity and reinforce stem‐like traits in cancer cells, thereby linking Hippo pathway deregulation to the acquisition of CSC properties (Fig. 2).

## 3 Hippo pathway and stemness in breast cancer

While the Hippo pathway plays a crucial role in normal mammary gland development, its dysregulation is a critical event in BC progression and development [50, 51, 52]. The oncogenic role of the Hippo pathway in BC was first demonstrated by Overholtzer and colleagues, who showed that YAP overexpression in non‐transformed mammary epithelial cells was sufficient to induce EMT and growth factor‐independent proliferation [53]. Subsequent studies using YAP/TAZ transgenic mouse models provided further evidence that these proteins can initiate tumorigenesis and reprogram primary cells into mammary stem cells [54, 55]. A wealth of subsequent studies has been instrumental in defining the involvement of dysregulated Hippo signaling in stemness [19, 56, 57, 58], as well as in stemness‐related processes such as mechanotransduction [59], distant dissemination [60, 61] and resistance to chemotherapies [62, 63].

In physiological conditions, YAP is associated with the core stem genes Octamer‐binding transcription factor 4 (OCT4) and Homebox protein NANOG (NANOG) promoters to regulate mouse embryonic stem cell self‐renewal [64]. Similarly, Cordenonsi and colleagues provided the first evidence of the role of Hippo pathway effectors in promoting stemness in BC. They observed higher YAP/TAZ levels in poorly differentiated/high‐grade breast tumors. Using Ras‐transformed MCF10A mammary cells, they confirmed higher TAZ and YAP mRNA and protein levels in BCSCs compared with their non‐stem counterpart. YAP/TAZ overexpression was associated with mammosphere‐forming ability and maintenance of the CD44^+^/CD24^−/low^ immunophenotype. At the molecular level, EMT promotes delocalization of the regulator of epithelial polarity, and TAZ inhibitor, Scribble planar cell polarity protein (Scribble). Released from Scribble inhibition, TAZ promotes the acquisition of CSC traits by sustaining survivin levels, inhibiting apoptosis, and promoting chemo‐resistance [19]. Likewise, YAP activates the transcription of mammary stem cell signature genes (e. g., SOX2, OCT4, NANOG) in BCSCs [56, 65]. Using an *in vivo* metastatic model that recapitulates early‐stage BC progression, Bartucci and colleagues demonstrated that BCSCs possess the ability to initiate metastasis. Transcriptomic analyses comparing metastasis‐promoting and non‐metastasis‐promoting cell populations identified TAZ as a central driver of BCSC‐mediated metastasis, chemo‐resistance, and tumorigenicity. Silencing TAZ in BCSCs markedly reduced their capacity for metastatic colonization and resistance to chemotherapy. Clinically, analysis of tumor samples from 99 BC patients revealed that high TAZ expression correlates with shorter disease‐free survival, suggesting that TAZ serves as an independent negative prognostic marker. Collectively, these findings establish TAZ as a critical regulator of BCSC‐driven metastasis [24].

Other mechanisms have been reported to contribute to YAP/TAZ stemness activity through the loss or downregulation of upstream regulators of the Hippo pathway. Tian and colleagues found that the Hippo pathway activator Rho GTPase‐activating protein (GAP) interacting with CIP4 homologs protein 1 (RICH1) is downregulated in BC specimens from the The Cancer Genome Atlas (TCGA) cohort compared with normal tissues. Mechanistically, the GAP RICH1 competes with Merlin for binding to Amot‐p80, thereby displacing the Hippo regulatory complex of Merlin and Amot and activating Hippo signaling. RICH1‐depleted BC cell lines exhibited increased levels of YAP and TAZ, resulting in enhanced stemness‐related features. A higher fraction of CD44^+^/CD24^−/low^ cells could also be sorted from these cell populations. Accordingly, RICH1‐depleted MCF10A normal breast cells showed the loss of epithelial traits [21].

**Fig. 3:**
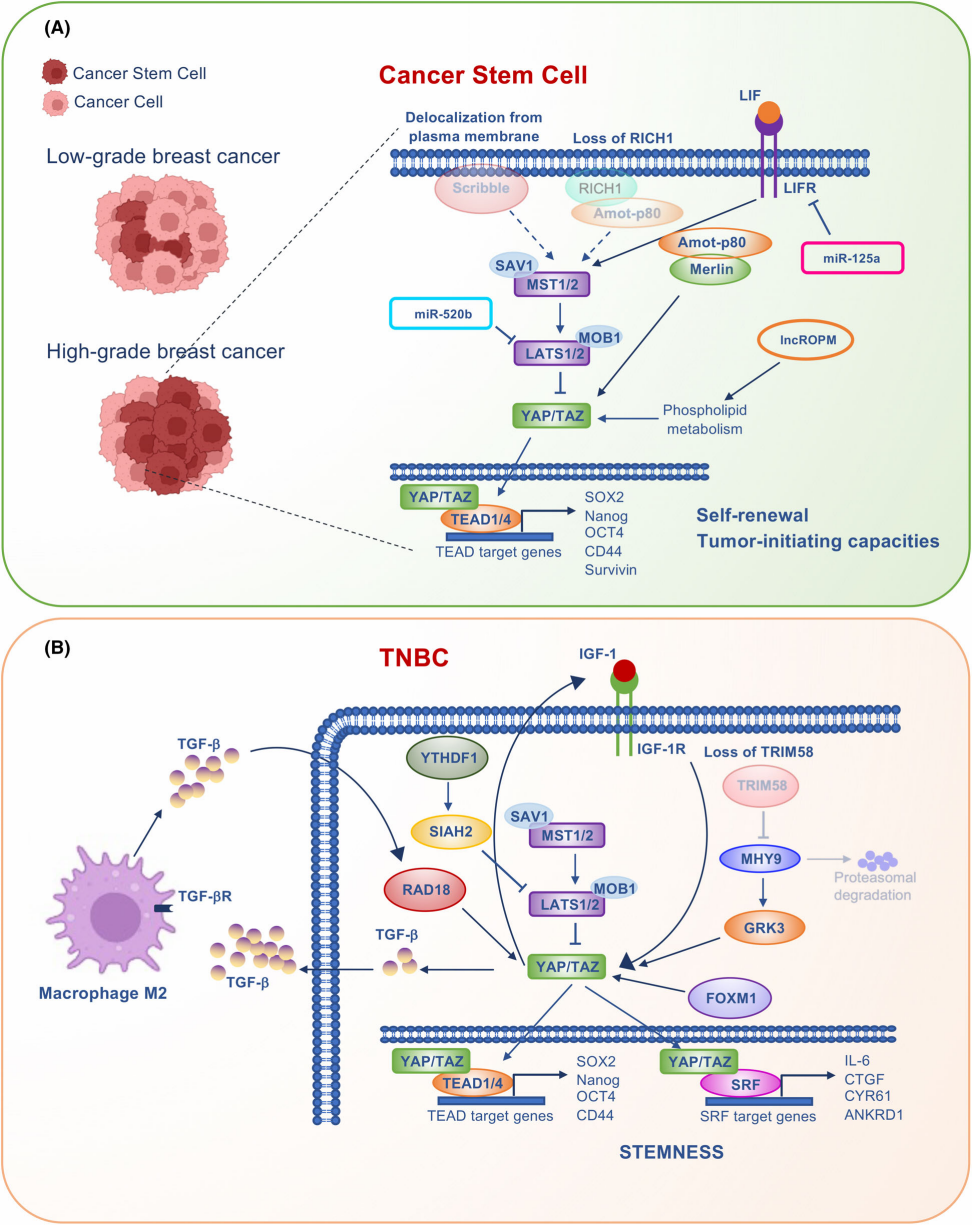
Mechanisms promoting YAP/TAZ‐related stemness in breast cancer. (A) High‐grade breast tumors are enriched in cancer stem cell (CSC) populations. In breast cancer stem cells (BCSCs), the loss or downregulation of upstream regulators of the Hippo pathway contributes to self‐renewal and tumor‐initiating capacity. Delocalization of Scribble from the plasma membrane and loss of RICH1 impair inhibition of Hippo signaling, thereby promoting TAZ translocation to the nucleus. miR‐520b enhances YAP‐dependent stemness by suppressing LATS2 expression, while miR‐125a targets LIFR, an inhibitor of Hippo signaling, leading to TAZ nuclear translocation and transcriptional activation of stemness‐related genes. The long non‐coding RNA lncROPM sustains phospholipid metabolism, which in turn activates YAP. (B) YAP supports the acquisition of stem‐like traits in triple‐negative breast cancer (TNBC) through its interaction with stemness‐related pathways, the tumor microenvironment (TME), metabolic reprogramming, and immune modulation. A positive feedback loop between IGFR and YAP promotes YAP translocation to the nucleus. Facilitating communication between the tumor and its microenvironment, the DNA damage response protein RAD18 triggers YAP‐dependent release of TGF‐β, which polarizes macrophages toward the M2 phenotype. TGF‐β released from M2 macrophages further activates RAD18, reinforcing the loop. The transcription factor FOXM inhibits YAP phosphorylation, promoting its nuclear translocation. Downregulation of TRIM58, observed in TNBC tissues compared to adjacent normal tissues, induces nuclear translocation of the transcriptional regulator MYH9, which in turn upregulates GRK3 expression, ultimately leading to YAP accumulation. SIAH2 induction by the N6‐methyladenosine‐binding protein YTHDF1 results in LATS2 inhibition and consequent YAP activation. Collectively, these mechanisms enhance the transcription of key stemness‐associated genes. As a TNBC‐specific mechanism, the SRF transcription factor recruits YAP to stemness‐related gene promoters, including IL6. IL6‐STAT3 signaling is a critical pathway mediating YAP‐induced stemness in TNBC. The partially transparent text used for some labels is intentional. This stylistic choice visually represents the loss or attenuation of the corresponding regulatory mechanisms within the Hippo pathway, as illustrated in the processes described above.

Also, several microRNAs (miRNAs) and long non‐coding RNAs (lncRNAs) have been connected with BCSCs through direct or indirect Hippo pathway modulation [66, 67, 68]. miR‐520b, which is often upregulated in BC and predicts poor prognosis, suppresses LATS2 expression, thus promoting YAP‐dependent stemness. Accordingly, miR‐520b is enriched in the CD44^+^/CD24^−/low^ cell lineage isolated from BC cell lines [66]. Similarly, miR‐125a induces TAZ activity and nuclear localization by targeting the Leukemia Inhibitory Factor Receptor (LIFR), an upstream activator of Hippo signaling. As a result, an array of stemness‐related genes is activated. A strong correlation between miR‐125a upregulation and LIFR downregulation has been described in clinical datasets, which also correlates with a poor prognosis. This mechanism is restricted to BCSCs and does not occur in the bulk cell population of BC samples [67]. Interestingly, miR‐520b has been reported to play protective roles in hepatoma cells, indicating context‐specific functions [69]. The lncRNA lncROPM is overexpressed in BCSCs and involved in the genesis and maintenance of these cells through stabilization of the metabolic enzyme PLA2G16, which leads to an increase in free fatty acids resulting in activation of YAP. By combining chemotherapy with the phospholipase A2 inhibitor giripladib, an effective reduction in BCSCs formation was obtained in both *in vitro* and *in vivo* models [68]. Thus, targeting Hippo‐related miRNA/lncRNA alone or in combination with chemotherapy was proposed as a way to target BCSCs. The mechanisms accounting for activation of YAP/TAZ‐related stemness in BC are illustrated in Fig. 3A.

### 3.1 The hippo pathway and stemness in triple‐negative breast cancer

Evidence indicates that YAP significantly contributes to the acquisition of CSC features in TNBC. Although YAP/TAZ activation is not exclusive to this subtype, recent analyses summarizing TCGA and METABRIC datasets indicate that their expression and activity tend to be higher in basal‐like/TNBC tumors compared with luminal or HER2‐positive subtypes [70]. This enrichment may help explain why many of the mechanistic links between Hippo signaling and stemness have been described in TNBC models, while remaining less well characterized in other BC contexts.

The mechanisms involved include interactions with stemness‐related pathways and with the tumor microenvironment (TME), metabolic reprogramming, and immune modulation. The serum‐response factor‐Interleukin‐6‐Signal Transducer and Activator of Transcription 3 (SRF‐IL6‐STAT3) signaling axis is a critical mediator of YAP‐induced stemness in TNBC. Indeed, SRF‐mediated recruitment of YAP to the promoters of stemness‐related genes is critical for YAP‐induced stemness, and current evidence suggests that this mechanism is restricted to the TNBC subtype [71]. Accordingly, the IL6‐STAT3 pathway is preferentially activated in CD44^+^CD24^−/low^ BC cells and in TNBC compared to other BC subtypes [72]. This enrichment is consistent with the higher burden of stem‐like and chemo‐resistant cells typically observed in TNBC, but it may also reflect subtype‐specific features of the inflammatory microenvironment, including sustained cytokine production and crosstalk with YAP/TAZ‐dependent transcriptional programs. From a translational standpoint, these findings raise the possibility that targeting the IL6–STAT3 axis, through IL‐6/IL6R blockade or downstream JAK/STAT inhibition, could help attenuate CSC‐associated traits in TNBC, although definitive clinical evidence for this strategy is still lacking, and alternative explanations, such as broader microenvironmental or genomic influences, cannot be excluded.

Other mechanisms have also been described in TNBC that ultimately result in YAP/TAZ‐mediated promotion of stemness. Using TNBC patient‐derived CSC models, Chan and colleagues demonstrated a crosstalk between the Hippo pathway and the stemness‐related Insulin‐like growth factor 1 receptor/Insulin‐like growth factor (IGF‐1R/IGF) pathway. Specifically, IGF‐1R sustains YAP overexpression and nuclear localization, which in turn induces IGF‐1 mRNA accumulation, establishing a positive feedback loop. Accordingly, high levels of IGF‐1 and YAP in patient specimens correlate with worse survival outcomes [73]. Similarly, the DDR protein RAD18 E3 Ubiquitin Protein Ligase (RAD18) induces YAP‐dependent stemness through LATS1 inhibition in TNBC cell models. By exploiting co‐culture models of TNBC cells with macrophages, the authors identified a mechanism of RAD18‐YAP‐mediated communication between the tumor and its microenvironment that sustains stemness in TNBC. In detail, TGF‐β secretion resulting from RAD18‐induced YAP activation in TNBC regulates macrophage polarization toward the M2 phenotype, which is associated with tumor progression and immune suppression. In turn, M2 macrophages produce TGF‐β that further activates RAD18 in TNBC in a positive feedback loop [48].

In TNBC cell models, the transcription factor Forkhead box M1 (FOXM1) has been involved in the inhibition of YAP phosphorylation, leading to its nuclear translocation and transcription of stemness‐related genes [74]. TNBC tissues and cells are characterized by lower expression of the E3 ubiquitin ligase Tripartite motif containing 58 (TRIM58) compared with adjacent normal tissues, and TRIM58 downregulation is associated with shorter survival. In CSCs derived from TNBC patients, TRIM58 downregulation was associated with YAP activation and stem cell traits. Indeed, TRIM58 downregulation induces the nuclear translocation of the transcriptional regulator Myosin Heavy Chain 9 (MYH9), which induces the G‐protein‐coupled receptor kinase 3 (GRK3) expression and ultimately results in YAP accumulation and stemness [75]. The N^6^‐methyladenosine‐binding protein YTH N6‐Methyladenosine RNA Binding Protein F1 (YTHDF1), highly expressed in TNBC cell models, was shown to play a crucial role in sustaining the expression of seven in absentia homology 2 (SIAH2), a Hippo pathway regulator that inhibits LATS2. This interaction promotes YAP/TAZ activation, driving the transcription of stemness‐related genes [76].

Although these mechanisms have been described in TNBC, the pathways involved are not known to be exclusive to this subtype. Instead, they likely reflect pathways that are particularly active or clinically relevant in basal‐like/TNBC tumors, consistent with the higher YAP/TAZ activity observed in these cancers. At the same time, the extent to which similar Hippo‐dependent mechanisms contribute to stemness or prognosis in other BC subtypes remains less well defined and represents an important area for future investigation. The mechanisms connecting YAP/TAZ to stem cell‐related features in TNBC are reported in Fig. 3B.

## 4 Hippo pathway between stemness and therapeutic resistance

### 4.1 Resistance to chemotherapy and radiotherapy

Resistance to chemotherapy and radiotherapy remains a significant challenge in BC treatment, particularly in TNBC. Current guidelines recommend chemotherapy as the mainstay treatment for TNBC, the most aggressive subtype of BC, in the absence of actionable biomarkers such as PD‐L1 expression or BRCA mutations. The standard chemotherapy regimens include taxanes (e.g., paclitaxel, docetaxel), anthracyclines (e.g., doxorubicin, epirubicin), and platinum salts (e.g., carboplatin).

The Hippo pathway has been implicated in acquired resistance to chemotherapy across various cancers, including colon cancer, hepatocellular carcinoma, and BC [77, 78, 79]. Lai and colleagues first demonstrated the involvement of the Hippo pathway in paclitaxel resistance, showing that elevated TAZ expression in TNBC cell lines correlates with reduced sensitivity to paclitaxel. At the mechanistic level, TAZ drives drug resistance by inducing the transcription of the oncogenes CYR61 and CTGF. Notably, silencing TAZ restores taxol sensitivity [77]. Anti‐tubulin agents primarily induce apoptosis by activating the cell cycle kinase cyclin‐dependent kinase 1 (Cdk1), which phosphorylates YAP at specific, Hippo pathway‐independent sites, leading to its inactivation. Mutations in these phosphorylation sites reduce apoptosis induced by anti‐tubulin drugs, suggesting that YAP is inactivated by Cdk1 phosphorylation. Moreover, in taxol‐resistant TNBC cell models, YAP was not phosphorylated following anti‐tubulin drug treatment [80].

YAP dysregulation has also been implicated in resistance to cisplatin in TNBC. In a cohort of metastatic and cisplatin‐resistant TNBC patients, Kim and colleagues identified an increased mutational frequency in the epigenetic regulator SMARCA4. The inactivation of SMARCA4 led to nuclear accumulation of YAP and subsequent transcription of target genes, promoting EMT and enhancing cisplatin resistance. Treatment of SMARCA4‐knockout TNBC cell models with the YAP inhibitor Verteporfin restored cisplatin sensitivity, providing evidence for a role of YAP in acquired resistance mechanisms [81].

Collectively, these findings highlight that targeting Hippo pathway dysregulation represents a promising therapeutic strategy for overcoming chemo‐resistance in TNBC. Drug repurposing has shown potential in this context: Yang and colleagues identified the antipsychotic drug chlorpromazine (CPZ) as effective in reducing BCSC properties in cell models. CPZ treatment suppresses YAP expression through proteasomal degradation, leading to diminished stemness features. Furthermore, in drug‐resistant cell models, a combination of CPZ with chemotherapy demonstrated significantly greater efficacy in reducing cell viability and stemness features compared to chemotherapy alone [82]. Next, post‐chemotherapy immunohistochemistry analysis of both ER‐positive and ER‐negative tumors revealed elevated expression of Type 1 Receptor Tyrosine Kinase‐Like Orphan Receptor (ROR1), a key regulator of cancer stemness. ROR1 accumulation was observed in cancer stem cells isolated from patient‐derived xenografts (PDX) and was associated with increased TAZ nuclear localization. Treatment with the anti‐ROR1 antibody cirmutuzumab reduced TAZ nuclear accumulation, attenuated stemness features, and impaired tumor engraftment in mice. Furthermore, combined administration of cirmutuzumab and paclitaxel demonstrated greater efficacy in reducing tumor size *in vivo* compared to individual drug [83].

Concerning radiotherapy, Liang and colleagues recently demonstrated that ionizing radiation in TNBC triggers upregulation of CD146, which forms a complex with Integrin β1 to inactivate LATS1 and drive YAP nuclear translocation. This cascade exacerbates abnormal DDR, enhances EMT, and amplifies stemness features, collectively contributing to radioresistance. Notably, targeting CD146 or Integrin β1—either alone or in combination—rescues radiosensitivity, offering a novel therapeutic avenue for TNBC [84]. Key examples of stemness‐related mechanisms of chemo‐resistance driven by Hippo pathway dysregulation are reported in Table 1.

**Table 1:** Key stemness‐related mechanisms of resistance to chemotherapies driven by Hippo pathway dysregulation.

Drug involved	Context and proposed mechanism	Strategy to overcome resistance	References
Taxol	In BC cell lines, elevated TAZ expression dictates reduced sensitivity to taxol by inducing the transcription of CYR61 and CTGF oncogenes	Administration of chemotherapy combined with chlorpromazine (which promotes YAP proteasomal degradation and diminishes stemness)	Lai D, Canc Res 2011 [77] Yang CE, Chem Biol Interact 2019 [82]
	In cell models, taxol‐activated Cdk1 inactivates YAP by phosphorylation at Hippo‐independent sites. This mechanism is impaired in taxol‐resistant TNBC cell lines	Mutation of YAP critical sites relieves its ability to block taxol‐induced apoptosis	Zhao Y, Canc Res 2014 [80]
Cisplatin	Metastatic, cisplatin‐resistant TNBC patients are enriched for mutations in the *SMARCA4* gene. *SMARCA4* inactivating mutations lead to YAP nuclear accumulation and activation of EMT	Co‐treatment with YAP inhibitor verteporfin	Kim J, Cancers 2021 [81]
Paclitaxel	Post‐therapy BC tissues are enriched for the stemness regulator ROR1. In CSCs isolated from BC PDXs, high ROR1 expression promotes TAZ nuclear accumulation	Combined administration of the anti‐ROR1 antibody cirmutuzumab and paclitaxel	Zhang S, PNAS 2019 [83]
Radiotherapy	In TNBC cell cultures, ionizing radiation promotes YAP‐driven EMT and stemness by upregulating CD146, which inhibits LATS1 enabling YAP nuclear translocation	Targeting CD146 or Integrin β1	Liang Y, Cancer Lett 2022 [84]

Due to the critical role of Hippo pathway dysregulation in the progression and therapeutic resistance across many tumors, targeting this pathway represents a promising therapeutic strategy. In addition, YAP1 overexpression is associated with early relapse in TNBC patients treated with chemotherapy, suggesting a significant role of YAP1 in chemo‐resistance. However, systemic YAP/TAZ inhibition may lead to severe toxicity due to the essential role of YAP/TAZ in maintaining homeostasis [85].

Several strategies have been proposed, encompassing disruption of YAP/TAZ‐TEAD interaction, inhibition of YAP/TAZ nuclear localization, targeting the palmitoylation pocket of TEADs and inhibition of upstream mechanical cues [86]. Verteporfin, a benzoporphyrin derivative that disrupts YAP‐TEAD interactions, has been shown to reduce BCSC populations and restore taxol sensitivity in TNBC cell models. Li and colleagues demonstrated that YAP1 inhibition by verteporfin leads to reduced cancer cell growth and proliferation and restores sensitivity to taxol in resistant TNBC cell lines [87]. Apigenin, a natural flavone, significantly inhibited the stem‐like properties of TNBC cells by disrupting YAP/TAZ‐TEAD interaction and downregulating stemness‐related genes [88]. Likewise, Zanconato and colleagues demonstrated that inhibiting the YAP/TAZ co‐activator Bromodomain Containing 4 (BRD4) with JQ1, a Bromodomain and Extra‐Terminal (BET) inhibitor, sensitizes YAP/TAZ‐addicted TNBC cell models *in vitro* [89].

Given that WNT and YAP signaling promote stemness in TNBC, Sulaiman and colleagues developed nanoparticles loaded with the Wnt inhibitor PRI‐724 and the YAP/mevalonate inhibitor simvastatin and assessed their efficacy in PDX models. Co‐administration of these nanoparticles with paclitaxel reduced paclitaxel‐induced CSC enrichment and effectively impaired the growth of paclitaxel‐resistant PDX tumors [90]. However, at present, therapeutic strategies combining Hippo pathway targeting with chemotherapy remain confined to the preclinical setting, where they have shown promising potential but have yet to be translated into clinical application.

### 4.2 Resistance to endocrine therapy

Endocrine therapy is the mainstay of treatment for hormone receptor (HR)‐positive, HER2‐negative BC, which accounts for approximately 70% of all BCs [91]. Tamoxifen, the first selective estrogen receptor modulator (SERM), was approved in the 1970s [92]. Along with SERMs, aromatase inhibitors are also drugs of choice for hormone‐sensitive BC [93].

The critical role of BCSCs in the onset of tamoxifen resistance is well established [15]. Among the mechanisms involved, the crosstalk between ERα and the stemness regulator SRY (Sex‐Determining Region Y)‐Box 2 (SOX2) has been elucidated by Zhang and colleagues. Estradiol stimulation of MCF7 BC cells prompts ERα localization to the miR‐140 promoter, resulting in its transcriptional silencing and subsequent SOX2 upregulation. Consistently, miR‐140 is significantly downregulated in BC patient samples compared to non‐tumorigenic counterparts [94]. Additional studies have demonstrated that ERα Ser118 phosphorylation plays a crucial role in both stemness acquisition and tamoxifen resistance [11, 12]. Accordingly, stem‐like subpopulations exhibit increased activity of MAPK and CDK7, upstream kinases that directly induce ERα Ser118 phosphorylation [95, 96]. Furthermore, Hippo pathway inactivation and YAP nuclear accumulation are key features accompanying the loss of the tumor suppressor WW Domain Containing Oxidoreductase (WWOX), which results in tamoxifen resistance in HR‐positive, HER2‐negative MCF7 cells. Notably, YAP depletion in WWOX‐knockdown cells restores tamoxifen sensitivity [97]. Consistently, WWOX downregulation enhances stemness in hormone‐sensitive cell models, while high WWOX expression in BC patients predicts favorable responses to tamoxifen therapy [98, 99].

Beyond tamoxifen‐specific mechanisms, several Hippo‐dependent processes have been implicated in broader endocrine therapy resistance, even though direct evidence for other SERMs/selective estrogen receptor degraders (SERDs) remains limited. These mechanisms primarily involve Hippo‐mediated modulation of ERα transcriptional activity, chromatin regulation, and metabolic reprogramming within the stem cell compartment. A hallmark of BCSCs is metabolic plasticity, characterized by constant switching between glycolysis and oxidative metabolism [100]. In BC cell models, YAP transcriptionally regulates the lncRNA BCAR4, a key driver of glycolysis implicated in endocrine resistance [101, 102].

Moreover, an intricate crosstalk between the Hippo and ER pathways has been documented. On the one hand, YAP directly represses the transcription of *ESR1* (the gene encoding ER) via vestigial‐like protein 3 (VGLL3)‐mediated recruitment of NCOR2/SMRT repressor to the *ESR1* super‐enhancer [103]. In a feedback regulatory loop, active ER signaling induces G‐protein‐coupled estrogen receptor (GPER) activation, leading to YAP and TAZ nuclear translocation and contributing to tamoxifen resistance [104, 105]. In addition, YAP can inhibit the ERα transcription program through a competitive TEAD‐dependent mechanism. Indeed, TEAD physically interacts with ERα to enhance ERα's promoter/enhancer binding, driving estrogen‐dependent transcription. Nuclear YAP competes with ERα for TEAD binding, leading to reduced ERα occupancy at target gene promoters/enhancers and proteasomal degradation of ERα, diminishing its transcriptional activity. Pharmacological inhibition of the Hippo/MST1/2 pathway suppressed tumor growth driven by hormone therapy‐resistant ERα mutants, suggesting that targeting the Hippo‐YAP‐TEAD signaling axis may offer a promising therapeutic strategy to overcome endocrine resistance associated with these mutations [106]. These insights also highlight potential therapeutic opportunities, as interventions that disrupt this Hippo‐ERα convergence, such as targeting the upstream kinases responsible for Erα ser118 phosphorylation or inhibiting YAP/TEAD activity, may help counteract stemness‐associated endocrine resistance. Key examples of stemness‐related mechanisms of resistance to endocrine therapy driven by Hippo pathway dysregulation are reported in Table 2.

**Table 2:** Key stemness‐related mechanisms of resistance to endocrine therapy driven by Hippo pathway dysregulation.

Drug involved	Context and proposed mechanism	Strategy to overcome resistance	References
Tamoxifen	In MCF7 cells, YAP nuclear accumulation accompanies WWOX loss, promoting stemness and tamoxifen resistance	YAP depletion in WWOX‐knockdown cells restores sensitivity	Li J, Exp Biol Med 2019(97) Li J, Exp Biol Med 2018 [98] Göthlin Eremo A, Oncol Reports 2013 [99]
	ER signaling through G‐protein coupled estrogen receptor (GPER) leads to YAP/TAZ nuclear translocation	Prolonged treatment of MCF‐7 cells with the GPR30 agonist G1 mimics long‐term tamoxifen exposure and enhances its agonistic effects	Ignatov A, Breast Cancer Res Treat 2010 [105]
Endocrine therapy	In BC cell cultures, YAP upregulates the lncRNA BCAR4, a driver of glycolysis, promoting tamoxifen resistance	Therapeutic delivery of locked nucleic acids (LNAs) targeting BCAR4	Zheng X, Embo J 2017 [101] Godinho M, J Cell Physiol 2011 [102]
	In MCF7 cells, YAP target gene VGLL3 recruits NCOR2/SMRT repressor to the *ESR1* super‐enhancer, inhibiting its transcription	The LATS inhibitor VT02956 represses *ESR1* expression and inhibits the growth of patient‐derived organoids	Ma S, Nat Commun 2022 [103]
	In ER+ cell lines, YAP competes with ERα for TEAD binding, reducing ERα activity and leading to its degradation	MST1/2 inhibition effectively suppresses tumor growth driven by hormone therapy‐resistant ERα mutations	Li X, Nat Commun 2022 [106]

Collectively, these findings highlight how Hippo signaling is involved in the biology of ER‐positive BC and suggest that targeting this pathway could represent a promising therapeutic approach for hormone‐resistant BC. Accordingly, combining endocrine therapy with Hippo pathway modulation improves outcomes in endocrine‐resistant BC [107]. Beyond direct strategies such as MST1/2 inhibition, indirect approaches have also been explored. Histone deacetylase inhibitors (HDACi) modulate Hippo pathway signaling in HR‐positive BC. In HR‐positive HER2‐negative BC cell lines, HDACi treatment led to transcriptional downregulation of YAP expression while activating a TEAD‐mediated transcriptional program, upregulating canonical Hippo pathway genes. Four of these TEAD‐target genes (*CCDC80*, *GADD45A*, *F3*, and *TGFB2*) were associated with improved survival in a cohort of HR‐positive BC patients [108]. Additionally, miRNAs targeting Hippo pathway components have been proposed to enhance BC cell sensitivity to endocrine therapy. By modulating YAP/TAZ activity, these miRNAs could influence response to tamoxifen or aromatase inhibitors [107].

### 4.3 Resistance to CDK4/6 inhibitors

The therapeutic approach for metastatic ER‐positive, HER2‐negative BC typically involves the combination of endocrine therapy with cyclin‐dependent kinase 4 and 6 (CDK4/6) inhibitors [109, 110, 111]. Recent studies have highlighted the role of the YAP/TAZ axis in mediating acquired resistance to CDK4/6i in ER‐positive breast cancer. Genomic analysis of CDK4/6i‐treated ER‐positive BC samples revealed that resistance is associated with the accumulation of loss‐of‐function (LoF) mutations in the tumor suppressor FAT Atypical Cadherin 1 (FAT1). These FAT1 LoF mutations inhibit the activity of MST1/2, a key component of the Hippo signaling pathway, leading to YAP and TAZ nuclear translocation. Once in the nucleus, YAP and TAZ localize on the CDK6 promoter, driving its uncontrolled transcription. This results in elevated CDK6 expression, which bypasses CDK4/6 inhibition and promotes cell cycle progression and resistance [112]. Park and colleagues uncovered a novel mechanism by which YAP overexpression contributes to resistance to CDK4/6i. They identified Mitogen‐Activated Protein Kinase Kinase Kinase 3 (MEKK3) as a key regulator of YAP stability. Through its kinase activity, MEKK3 phosphorylates YAP at serine 405, preventing its translocation to lysosomes for degradation. The stabilization of YAP leads to its nuclear accumulation and transcriptional activation of pro‐survival and stemness genes, ultimately driving resistance to CDK4/6i. Importantly, silencing or pharmacological inhibition of MEKK3 effectively reduced YAP levels and restored sensitivity to CDK4/6i, highlighting MEKK3 as a potential therapeutic target for overcoming resistance [113].

### 4.4 Resistance to HER2‐targeted therapies

HER2 amplification occurs in 15–20% of BCs and is associated with an aggressive phenotype. Nevertheless, the development of HER2‐directed therapies revolutionized the management of HER2‐positive BC patients. The approval of trastuzumab, a HER2‐targeting monoclonal antibody, by the US FDA in 1998 marked a pivotal milestone in treatment, dramatically improving outcomes for HER2‐positive patients [114]. Subsequently, various HER2‐directed agents have been developed, significantly improving the prognosis of HER2‐positive BC patients [115]. However, the widespread availability of targeted agents administered sequentially has raised concerns regarding cross‐resistance [116] which, along with innate and acquired resistance, remains a critical challenge in the treatment of these patients.

The involvement of Hippo pathway dysregulation in CSC traits acquisition and escape from HER2‐targeted agents is supported by experimental studies. Phosphoproteomic and transcriptomic studies identified differential expression of YAP1 and TEAD2 genes in trastuzumab‐resistant cell models generated *in vitro* compared to their non‐resistant counterparts. From a mechanistic standpoint, YAP1 translocates into the nucleus and interacts with TEAD2 to transcriptionally regulate target genes linked to stemness acquisition, metastatization, and tumor relapse. Notably, the YAP inhibitor verteporfin restored trastuzumab sensitivity in resistant cells [117]. YAP and TAZ inhibition, achieved via pharmacological inhibition or proteins downregulation by siRNA, rescued trastuzumab efficacy in trastuzumab‐resistant cell cultures [118]. In both studies, YAP/TAZ and/or TEAD2 enrichment was observed in tissue samples of trastuzumab‐treated patients who experienced disease recurrence. Accordingly, emerging evidence suggests a possible association between TAZ expression and response to HER2‐targeted neoadjuvant therapy in breast cancer, although findings remain variable across studies and warrant further investigation in larger, subtype‐specific cohorts [119, 120].

YAP1 activation has also been described in BC cell models exposed to the antibody‐drug conjugate (ADC) Trastuzumab‐emtasine (T‐DM1). In this context, YAP mediates overexpression of the stemness‐associated gene ROR1 and induces self‐renewal of CSCs, leading to therapeutic resistance. Silencing of ROR1 and YAP1 reduced CSCs properties and rescued T‐DM1 sensitivity [121].

A critical route through which YAP/TAZ dictate pharmacological resistance in BC involves mechanotransduction. Mechanical stimuli (e.g., matrix stiffness) are sensed by YAP/TAZ and transduced in transcriptional programs that increase CSCs populations and promote stem‐like phenotypes, alongside other pro‐oncogenic processes [122]. These signals critically impair drug efficacy, accelerating therapeutic resistance. For instance, increased matrix stiffness drives resistance to the anti‐HER2 agent lapatinib through YAP and TAZ upregulation, whereas YAP/TAZ knockdown or treatment with verteporfin restored lapatinib sensitivity even in stiff matrices [123].

These observations indicate that Hippo‐driven stemness plays critical roles in limiting the efficacy of HER2‐targeted therapies and suggest that tackling YAP/TAZ holds the potential to improve the efficacy of anti‐HER2 agents. Key examples of stemness‐related mechanisms of resistance to HER2‐targeted therapies driven by Hippo pathway dysregulation are reported in Table 3.

**Table 3:** Key stemness‐related mechanisms of resistance to HER2‐targeted therapies driven by Hippo pathway dysregulation.

Drug involved	Context and proposed mechanism	Strategy to overcome resistance	References
Trastuzumab	In patients, YAP/TAZ and TEAD2 enrichment is associated with relapse following trastuzumab; in trastuzumab‐resistant cell models, YAP1 and TEAD2 activate transcription of stemness‐related and pro‐oncogenic genes.	Co‐treatment with YAP inhibitor verteporfin	González‐Alonso P, Cancers 2020 [117] Yuan JQ, Front Pharmacol 2020 [118]
T‐DM1	In BC cells, exposure to T‐DM1 induces YAP‐dependent expression of ROR1, enhancing CSC self‐renewal and promoting resistance	Silencing of ROR1 and YAP1 restores T‐DM1 sensitivity	Islam SS, EBioMed. 2019 [121]
Lapatinib	In HER2+ BC cells, stiffness‐inducedYAP/TAZ upregulation enhances CSC traits and impairs drug efficacy	YAP/TAZ knockdown or Verteporfin	Safei S, Cancer Cell Int 2023 [122] Lin CH, Mol Biol Cell 2015 [123]

Preclinical evidence supports the potential benefit of combining YAP/TAZ inhibitors with HER2‐targeted therapies to overcome resistance in BC. The non‐allosteric dihydrobenzofuran‐based inhibitor IAG933 disrupts YAP/TAZ‐TEAD complex formation by competitively binding to TEADs. In preclinical cell models, IAG933 exhibited dose‐dependent synergy with the HER2‐targeting agent lapatinib. Additionally, in a HER2‐amplified xenograft mouse model, the combination of IAG933 and trastuzumab led to complete tumor regression [124]. Currently, IAG933 is undergoing phase 1 clinical evaluation as a monotherapy for solid tumors (NCT04857372).

### 4.5 Immune remodeling and implications for immunotherapy

Emerging evidence suggests that Hippo pathway dysregulation may influence antitumor immunity in BC, although direct links to immune checkpoint inhibitor (ICI) resistance remain limited. Several studies indicate that YAP activity can modulate the tumor immune microenvironment (TIME) through effects on tumor‐associated macrophages (TAMs). In models of TAMs cultured with conditioned media from mouse BC cells, YAP/STAT3 signaling promotes macrophage polarization toward an M2‐like phenotype, which is associated with reduced CD8^+^ T‐cell activity and an immunosuppressive milieu [125]. Additional work in TNBC has shown that tumor‐derived signals can stabilize YAP in macrophages via OUT Deubiquitinase 5 (OTUD5)‐dependent deubiquitination, further enhancing M2 polarization and supporting tumor progression [126]. M2‐polarized TAMs, in turn, have been reported to promote EMT and stemness features in TNBC cells. Mechanistically, M2 TAMs secrete the chemokine (C‐C motif) ligand 2 (CCL2), which binds to C‐C chemokine receptor type 2 (CCR2) on BC cells, activating the PI3K/Akt/β‐catenin pathway [127]. Additionally, M2‐secreted IL‐6 activates STAT3 signaling in BC cells, leading to increased expression of stemness markers such as SOX‐2, OCT4, and NANOG, further enriching the CSC population [128].

Collectively, these findings point to a potential role for YAP in shaping macrophage‐mediated immune suppression and CSC enrichment, suggesting that YAP‐targeting strategies may have implications not only for stemness but also for modulating antitumor immunity. Likewise, approaches aimed at disrupting IL‐6/STAT3 signaling or reprogramming M2‐polarized macrophages may represent additional avenues for future investigation.

## 5 Current Hippo‐targeting strategies: From preclinical models to clinical translation

Despite the central role of Hippo pathway dysregulation in cancer stemness and therapeutic resistance, direct pharmacological targeting of this pathway has proven challenging. This difficulty largely stems from the lack of enzymatic activity of the downstream effectors YAP and TAZ and from their essential roles in normal tissue homeostasis. As a result, most therapeutic strategies have focused on disrupting YAP/TAZ transcriptional activity, interfering with their interaction with TEAD transcription factors, or targeting upstream regulatory mechanisms that govern YAP/TAZ localization, stability, and activity. Below, current direct and indirect Hippo‐targeted anti‐cancer therapeutic strategies under clinical evaluation in breast are discussed.

### 5.1 Direct hippo‐targeted agents

One of the most extensively investigated approaches involves disruption of the YAP/TAZ‐TEAD interaction. Verteporfin, a benzoporphyrin derivative originally approved for photodynamic therapy in ocular diseases, was the first compound reported to inhibit YAP‐TEAD binding [129]. In preclinical cancer models, verteporfin suppresses YAP/TAZ‐TEAD transcriptional activity, reduces cell proliferation and stem‐like properties, and impairs expression of oncogenic downstream target genes. In BC cells, verteporfin inhibits YAP‐TEAD interaction and downregulates canonical target genes such as CYR61 and CTGF, inducing apoptosis across multiple molecular subtypes [130, 131]. However, its limited specificity and unfavorable pharmacokinetic profile have restricted its clinical applicability as a Hippo‐targeted therapeutic agent.

More recently, therapeutic efforts have shifted toward direct targeting of TEAD transcription factors themselves [132]. TEAD proteins require autopalmitoylation for their stability and transcriptional activity, and small molecules capable of binding the TEAD palmitoylation pocket have emerged as a promising drug class. Among these, VT3989, a pan‐TEAD autopalmitoylation inhibitor, has advanced to Phase I/II clinical trials in patients with advanced solid tumors harboring Hippo pathway alterations [133]. IK‐930, a TEAD1‐selective inhibitor, has also been evaluated in early‐phase clinical studies, although its development has been limited by modest clinical activity reported to date [134]. Additional palmitoylation pocket inhibitors under investigation include ISM6331, a potent pan‐TEAD inhibitor that entered Phase I clinical testing in late 2024 [135]; SW‐682, currently undergoing early‐phase clinical evaluation [136], and BPI‐460372 [137]. Although clinical data for these agents remain preliminary, their development collectively underscores the therapeutic promise of targeting TEAD transcriptional activity.

Beyond palmitoylation pocket inhibitors, several additional TEAD‐directed compounds are in preclinical or early clinical development for solid tumors, reflecting growing interest in this therapeutic axis. Among these, IAG933 is a non‐allosteric dihydrobenzofuran‐based inhibitor that disrupts YAP/TAZ–TEAD complex formation by competitively binding TEAD proteins and preventing transcriptional activation. Preclinical studies have demonstrated that IAG933 leads to eviction of YAP from chromatin, suppression of Hippo‐dependent transcriptional programs, and potent antiproliferative activity in Hippo‐dependent cancer models, inducing tumor regression in xenograft models [124]. Importantly, IAG933 has progressed to phase I clinical evaluation in patients with advanced solid tumors, representing one of the first clinically actionable strategies directly targeting Hippo pathway transcriptional output. Although efficacy data are not yet available, this trial provides proof‐of‐concept for the translational potential of Hippo pathway inhibition. BGC‐515, a recently developed TEAD inhibitor, has also entered early clinical testing for metastatic mesothelioma, epithelioid hemangioendothelioma, and other solid tumors [138], while ODM‐212, a pan‐TEAD inhibitor, is currently being evaluated in patients with advanced solid malignancies [139].

In addition to small‐molecule inhibitors, alternative direct Hippo‐targeted approaches are emerging. ION537, a YAP1‐directed antisense oligonucleotide, suppresses YAP1 expression at the mRNA level and has shown antitumor activity in preclinical models of YAP‐driven cancers, leading to its evaluation in early‐phase clinical studies [140].

Direct Hippo‐targeted agents currently under clinical investigation are summarized in Table 4. Collectively, these efforts highlight the rapid evolution of Hippo‐targeting strategies, with TEAD inhibition representing the most clinically advanced approach to date. Although the majority of these compounds remain in early‐stage development, their progression into clinical testing marks an important step toward therapeutically exploiting Hippo pathway vulnerabilities in cancer, including BC contexts characterized by YAP/TAZ‐driven stemness and resistance to therapy.

**Table 4:** Current investigational therapeutic strategies directly targeting the Hippo pathway.

Mechanism of action	Agent	Clinical trial	Phase	Trial role	Area of investigation	References
TEAD palmitoylation inhibitor	VT3989	NCT04665206	I/II, ongoing	Monotherapy and combination (Nivolumab, Ipilimumab, or Osimertinib)	Refractory mesothelioma and other metastatic solid tumors with NF2 mutations or YAP/TAZ gene rearrangements	Yap TA, Nat Med 2025 [133]
	IK‐930	NCT05228015	I, discontinued due to limited activity	Monotherapy	Solid Tumors	Young N, Cancer Res 2023 [134]
	ISM6331	NCT06566079	I, ongoing	Monotherapy	Advanced or metastatic malignant mesothelioma or other solid tumors	Li Q, Cancer Res 2024 [135]
	SW‐682	NCT06251310	I, ongoing	Monotherapy	Metastatic or unresectable advanced solid tumors	Chen L, Cancer Res 2023 [136]
	BPI‐460372	NCT05789602	I, ongoing	Monotherapy	Solid Tumors	Han X, Cancer Res 2024 [137]
YAP/TAZ‐TEAD complex inhibitor	IAG933	NCT04857372	I, ongoing	Monotherapy	Mesothelioma, NF2/LATS1/LATS2 mutated tumors and tumors with functional YAP/TAZ fusions	Chapeau EA, Nat Cancer 2024 [124]
	BGC‐515	NCT06452160	I, ongoing	Monotherapy	Advanced solid Tumors	Guo et al, Cancer Res 2023 [138]
Pan‐TEAD inhibitor	ODM‐212	NCT06725758	I/II, ongoing	Monotherapy	Advanced solid Tumors	ISRCTN Registry 2024 [139]
YAP1 Antisense Oligonucleotide	ION537	NCT04659096	I, discontinued	Monotherapy	Advanced solid Tumors	Macleod et al, Cancer Res 2021 [140]

### 5.2 Indirect modulators of hippo signaling

Parallel strategies aim to indirectly suppress YAP/TAZ activity by targeting upstream regulators. While none of these agents were originally developed to target the Hippo pathway, accumulating preclinical and translational evidence indicates that they indirectly modulate YAP/TAZ signaling through biomechanical, receptor‐mediated, or metabolic routes.

Among inhibitors of mechanotransduction pathways, focal adhesion kinase (FAK) represents a key mediator of stiffness‐induced YAP activation. In preclinical models, FAK inhibition disrupts YAP/TAZ nuclear localization and transcriptional output, providing a strong biological rationale for their investigation as indirect Hippo‐modulating agents [141, 142]. Several FAK inhibitors, including SIGX1094 and IN10018, are currently under clinical evaluation in patients with advanced solid tumors including breast cancer, where YAP/TAZ‐driven phenotypes such as stemness and chemo‐resistance are mechanistically implicated [143, 144]. Notably, in February 2025 the U.S. FDA granted Fast Track Designation to SIGX1094 as a targeted therapy for diffuse gastric cancer, highlighting the clinical promise of this class of agents. IN10018 remains a cornerstone of clinical evaluation, with 2025 data emphasizing its potential to overcome drug resistance in KRAS‐mutant cancers [144].

G‐protein‐coupled receptors (GPCRs) function as upstream signaling “switches” that dynamically regulate YAP/TAZ activity. Therapeutic agents targeting GPCR pathways include ONC201, a first‐in‐class imipridone that acts as a selective antagonist of dopamine receptor D2 (D2R) and recently received FDA approval for H3K27M‐mutant diffuse midline glioma [145, 146, 147]. Similarly, the angiotensin II type 1 receptor (AT1R) inhibitor losartan has been explored in oncologic settings primarily for its effects on tumor fibrosis, tissue tension, and stromal remodeling, with secondary consequences indicating attenuation of YAP/TAZ activity through reduced mechanical stress [148]. Although these agents have not yet been evaluated in breast cancer, their capacity to modulate upstream Hippo signaling provides a rationale for future investigation, particularly in YAP/TAZ‐driven and therapy‐resistant BC subtypes.

Finally, YAP/TAZ activity is highly sensitive to the cellular metabolic state, particularly to the mevalonate pathway, which generates geranylgeranyl pyrophosphate (GGPP) required for Rho GTPase activation and YAP/TAZ nuclear localization [149, 150]. Metabolic interventions targeting this axis are therefore under active investigation. 3‐hydroxy‐3‐methyl‐glutaryl coenzyme A (HMG‐CoA) reductase inhibitors such as simvastatin and atorvastatin have been shown to suppress YAP/TAZ activity by depleting GGPP pools and impairing RhoA signaling [151, 152]. Multiple clinical trials are evaluating statins as adjunctive therapies in BCs, where YAP/TAZ‐driven transcriptional programs contribute to tumor progression and therapy resistance. In parallel, metformin indirectly impinges on Hippo signaling through activation of AMPK, which promotes inhibitory phosphorylation of YAP at Ser127 and its cytoplasmic sequestration [153, 154]. Metformin is widely used in window‐of‐opportunity clinical trials across several tumor types to assess its impact on epithelial–mesenchymal transition and cancer stemness, processes closely linked to YAP/TAZ activity. Indirect Hippo pathway modulators currently under clinical investigation in BC patients are summarized in Table 5.

**Table 5:** Current investigational therapeutic strategies indirectly modulating the Hippo pathway.

Mechanism of action	Agent	Clinical trial	Phase/status	Trial role	Area of investigation	References
FAK inhibitor	SIGX1094R	NCT06739291	I Active, recruiting	Monotherapy	Advanced Solid Tumors	Zhang H, Cancer Res 2025 [143]
	IN10018	NCT06166836	Ib/II Active, recruiting	Combination (KRAS^G12C^ inhibitor D‐1553)	KRAS^G12C^‐mutant solid tumors	Zhang B, Cancer Res 2025 [144]
HMG‐CoA reductase inhibitor	Simvastatin	NCT03324425	II Active, not recruiting	Combination (Dual HER2‐blockade)	Breast cancer	Sorrentino G, Nat Cell Biol 2014 [151] Lin Q, Mol Cell Oncol [152]
		NCT05464810	I Active, recruiting	Combination (letrozole)	HR+, HER2‐ breast cancer	
		NCT05550415	II Active, recruiting	Combination (chemotherapy)	Triple‐negative breast cancer	
	Atorvastatin	NCT04601116	III Active, recruiting	Combination (standard neoadjuvant therapy)	Early breast cancer	
AMPK activator	Metformin	NCT05023967	Phase IIb Active, not recruiting	Combination (nightly fasting)	Early breast cancer	Liu J, J Cell Mol Med 2020 [153] Xu Y, J Chemother 2023 [154]
		NCT07098299	I Active, recruiting	Combination (chemotherapy and/or immunotherapy)	Solid malignancies	

### 5.3 Epigenetic agents modulating hippo signaling

Epigenetic regulation represents a critical layer of YAP/TAZ‐driven oncogenic signaling and offers complementary therapeutic opportunities, particularly in contexts where direct Hippo pathway inhibition is limited or adaptive resistance emerges. In BC, YAP/TAZ function is tightly coupled to chromatin state and transcriptional co‐factor availability, making epigenetic modulators especially relevant for targeting YAP/TAZ‐dependent stemness, plasticity, and therapy resistance [155, 156, 157].

Among the most advanced strategies, BET bromodomain inhibitors disrupt BRD4‐dependent transcriptional programs that cooperate with YAP/TAZ at super‐enhancer regions. BRD4 physically and functionally interacts with YAP/TAZ to sustain transcription of genes involved in proliferation, EMT, and cancer stem cell maintenance [89]. In preclinical BC models, BET inhibition effectively phenocopies YAP/TAZ inactivation by disrupting the physical association between YAP/TAZ and BRD4 at distal enhancers. This blockade suppresses the YAP/TAZ‐driven transcriptional program responsible for stem‐like traits and phenotypic plasticity and reverses acquired resistance to CDK4/6 inhibitors and conventional chemotherapies [89, 158]. Among BET inhibitors, ZEN‐3694 is currently under clinical evaluation in solid tumors, and its ability to selectively impair YAP/TAZ‐addicted transcriptional programs supports its potential repurposing for YAP/TAZ‐driven BC subtypes [159]. BMS‐986158 (also known as Trotabresib) is a potent, small‐molecule inhibitor of BET proteins that has shown activity in BC models, particularly TNBC [160].

Another emerging axis involves the SWItch/Sucrose Non‐Fermentable (SWI/SNF) chromatin remodeling complex, which acts as a gatekeeper of YAP/TAZ activity. In normal epithelial cells, SWI/SNF components, such as ARID1A, can restrain YAP/TAZ by limiting their chromatin accessibility or TEAD binding. Loss‐of‐function mutations in SWI/SNF subunits, which occur in a subset of BCs and are enriched in aggressive phenotypes, release this constraint and enhance YAP/TAZ transcriptional output [161, 162]. Pharmacological strategies aimed at modulating SWI/SNF function or exploiting synthetic lethal interactions in SWI/SNF‐deficient tumors are therefore being explored as indirect means to suppress YAP/TAZ‐driven oncogenic programs [163, 164, 165, 166].

Finally, YAP/TAZ‐driven transcription critically depends on recruitment of the Super Elongation Complex (SEC), including cyclin‐dependent kinase 9 (CDK9), to promote RNA polymerase II–mediated transcriptional elongation. CDK9 inhibitors have emerged as a strategy to blunt the downstream transcriptional output of the YAP/TAZ–TEAD complex without directly targeting its formation. In BC models, CDK9 inhibition suppresses YAP/TAZ‐dependent gene expression, reduces cancer stem cell populations, and sensitizes tumors to cytotoxic and targeted therapies, providing a strong rationale for clinical exploration of this approach [167, 168, 169]. Epigenetic drugs currently under clinical investigation in BC patients are summarized in Table 6.

**Table 6:** Current investigational epigenetic therapeutic strategies targeting the Hippo pathway.

Mechanism of action	Agent	Clinical trial	Phase/status	Trial role	Area of investigation	References
BET inhibitor	ZEN‐3694	NCT05372640	I Active, recruiting	Combination (amebaciclim)	NUT carcinoma/breast cancer/solid cancer	Aftimos P, J Clin Oncol 2022 [159]
		NCT05422794	Ib Active, recruiting	Combination (pembrolizumab + paclitaxel)	Triple‐negative breast cancer	
		NCT05327010	II Active, recruiting	Combination (talazoparib)	Selected solid tumors	
		NCT05053971	I/II Active, recruiting	Combination (entinostat)	Solid tumors	
		NCT05803382	I Active, recruiting	Combination (capecitabine)	Solid tumors	
		NCT04840589	I/Ib Active, recruiting	Combination (nivolumab +/− ipilimumab)	Solid tumors	
	BMS‐986158	NCT02419417	Phase I/IIa Completed	Monotherapy and combination (nivolumab)	Solid tumors	Shapiro GI, J Clin Oncol 2020 [160]
SWI/SNF‐targeted agent	FHD‐909	NCT06561685	I Active, recruiting	Monotherapy and combination (anti‐cancer agents)	SMARCA4‐deficient solid tumors	Lee JY, Mol Cancer Ther 2024 [163]
	PRT3789	NCT06682806	II Active, not recruiting	Combination (pembrolizumab)	SMARCA4‐deficient solid tumors	Hilse M, Cancer Res 2026 [164]
	Ceralasertib	NCT05582538	II Active, recruiting	Priming (followed by durvalumab + paclitaxel)	Triple‐negative breast cancer	Ring A, Clin Cancer Res 2023 [165] Wilson Z, Cancer Res 2022 [166]
		NCT04090567	II Active, recruiting	Combination (olaparib)	BRCA‐mutated breast cancer	
		NCT02264678	I/Ib Active, not recruiting	Combination (olaparib)	BRCA‐mutated breast cancer/HRR‐mutated triple‐negative breast cancer	
		NCT04704661	I Active, recruiting	Combination (T‐Dxd)	Solid tumors with HER2 protein/gene	
CDK9i	CYC065	NCT02552953	Phase I Completed	Monotherapy	Solid tumors	Brisard D, Oncotarget 2018 [168] Soosainathan A, Cancer Res 2024 [169]
		NCT04983810	I/II Active, recruiting	Monotherapy	HER2 refractory/HR + HER2−/triple‐negative breast cancer	

Collectively, epigenetic Hippo‐targeted strategies underscore the feasibility of targeting YAP/TAZ‐driven transcriptional addiction through chromatin and transcriptional co‐factor modulation. Although most of these approaches remain in early clinical or late preclinical development, their mechanistic relevance to BC stemness and therapeutic resistance positions them as promising candidates for future clinical trials, either as monotherapies in YAP/TAZ‐high tumors or in rational combinations with direct TEAD inhibitors, endocrine therapy, or chemotherapy.

## 6 Conclusions

The Hippo pathway has emerged as a crucial regulator of stemness‐related mechanisms in BC, particularly in TNBC. Hippo dysregulation leads to enhanced activity of YAP and TAZ, which drive the maintenance of BCSCs, EMT, and resistance to conventional therapies [19, 20, 21, 22, 23, 24]. The intricate interplay between Hippo signaling and other oncogenic pathways further underscores its role in BC progression and heterogeneity. Additionally, studies have indicated that Hippo pathway dysfunction increases tumor plasticity, enabling cancer cells to adopt stem‐like properties and evade immune surveillance [125, 126, 127, 128]. This adaptability not only enhances metastatic potential but also allows tumors to survive under hostile microenvironmental conditions. Achieving a deeper understanding of these mechanisms is key for developing novel therapeutic strategies restoring Hippo pathway functional integrity.

YAP/TAZ activation plays a central role in sustaining BCSC self‐renewal and tumor‐initiating potential, a finding particularly significant in TNBC. The ability of YAP/TAZ to drive BCSC expansion and survival contributes to increased metastatic potential and resistance to standard treatments, such as chemotherapy [80, 81]. Moreover, YAP/TAZ activation enhances cellular plasticity and consequently enables tumor cells to transition between different phenotypic states, further complicating therapeutic intervention. Targeting these mechanisms could provide new avenues for TNBC treatment.

Despite substantial progress, challenges remain in effectively targeting the Hippo pathway for therapeutic intervention. Current strategies aimed at inhibiting YAP/TAZ activity, including small‐molecule inhibitors and RNA‐based approaches, have shown promise in preclinical models [85, 86, 87, 88, 89, 90], but concerns regarding specificity, off‐target effects, tolerability, and long‐term toxicity remain unresolved. These limitations highlight the need to explore alternative points of intervention within the pathway. Upstream regulators such as MST1/2 and LATS1/2, as well as downstream components including TEAD transcription factors, represent attractive targets, particularly given the recent development of TEAD palmitoylation inhibitors that disrupt YAP/TAZ–TEAD transcriptional activity. Furthermore, the role of the tumor microenvironment in modulating Hippo signaling and BCSC maintenance warrants tailored investigations.

A major concern is the development of resistance mechanisms linked to Hippo pathway alterations. BC cells can exploit YAP/TAZ activity to evade targeted therapies and adapt to unfavorable conditions, including hypoxia, metabolic stress, and immune surveillance. The ability of YAP/TAZ to promote EMT and sustain a stem‐like phenotype further enhances tumor plasticity, enabling cancer cells to escape the cytotoxic effects of chemotherapy, radiotherapy, and even emerging immunotherapies. Moreover, crosstalk between Hippo signaling and other oncogenic pathways, such as PI3K/Akt, Wnt/β‐catenin, and TGF‐β, facilitates adaptive resistance, allowing tumors to shift dependency toward alternative survival mechanisms [46, 47, 48, 127]. This highlights the need for combination therapies that simultaneously target Hippo and partner oncogenic avenues to prevent therapeutic escape. For instance, co‐targeting YAP/TAZ with MEK inhibitors or immune checkpoint blockade has shown promise in preclinical models, suggesting that disrupting compensatory signaling pathways may enhance treatment efficacy [113]. Additionally, epigenetic modulators that restore Hippo pathway integrity or inhibit downstream transcriptional programs may provide novel therapeutic opportunities [89, 108]. Given the complexity of resistance mechanisms, future research should focus on identifying biomarkers predictive of YAP/TAZ‐driven resistance, enabling a more personalized therapeutic approach.

In conclusion, the Hippo pathway represents a pivotal regulatory axis in BC stemness and therapy resistance. Its dysregulation promotes tumor heterogeneity and treatment failure across multiple BC subtypes, not only TNBC but also HR‐positive and HER2‐enriched disease, where Hippo signaling intersects with endocrine resistance, HER2‐driven plasticity, and immune remodeling. Continued efforts to develop therapeutics targeting upstream regulators, YAP/TAZ–TEAD transcriptional complexes, and cooperating oncogenic pathways, either alone or in combination with existing treatments, will be essential to improving patient outcomes.

## Conflict of interest

The authors declare no conflict of interest.

## Author contributions

GS, GB, and MMS conceptualized and wrote the paper. GS, AP, SS, LC, and DM prepared the figures and tables. GB and MMS reviewed and edited the manuscript. PV, TA, LF, and EK visualized the manuscript. MMS supervised and wrote the final version of the manuscript. All authors read and agreed on the final version of the review.
